# Length of hospital stay after uncomplicated gastrectomy in the Netherlands: a nationwide cohort study

**DOI:** 10.1007/s00464-025-12103-7

**Published:** 2025-10-06

**Authors:** Maurits R. Visser, Daan M. Voeten, Suzanne S. Gisbertz, Jelle. P. Ruurda, Mark I. van Berge Henegouwen, Richard van Hillegersberg, Dutch Upper Gastrointestinal Cancer Audit (DUCA) Group, Boudewijn van Etten, J. Heisterkamp, Sjoerd M. Lagarde, Misha D. P. Luyer, Grard A. P. Nieuwenhuijzen, J. P. Pierie, Johanna W. van Sandick, Peter D. Siersema, Marije Slingerland, Meindert N. Sosef, Edwin S. van der Zaag

**Affiliations:** 1 Department of Surgery, University Medical Center Utrecht, University of Utrecht, Room G04-228, Heidelberglaan 100, 3584 CX Utrecht, The Netherlands; 2 Scientific Bureau, Dutch Institute for Clinical Auditing, Leiden, The Netherlands; 3 Department of Surgery, Amsterdam UMC Location Vrije Universiteit, Amsterdam, The Netherlands; 4 Department of Surgery, Amsterdam UMC Location University of Amsterdam, Amsterdam, The Netherlands; 5 Cancer Treatment and Quality of Life, Cancer Center Amsterdam, Amsterdam, The Netherlands

**Keywords:** Gastric carcinoma, Gastrectomy, Length of stay, Readmission

## Abstract

**Background:**

Longer length of hospital stay (LOS) has an impact on patient lives and hospital costs. As 60%–70% of gastrectomies are uncomplicated, identifying factors influencing LOS after uncomplicated gastrectomy could help reduce LOS. This study examined hospital variation in LOS and its association with readmission after uncomplicated gastrectomy.

**Methods:**

Patients undergoing gastrectomy for gastric cancer in the Netherlands were included from the Dutch Upper Gastrointestinal Cancer Audit (DUCA). Patients with any complications (Gastric Complications Consensus Group definitions) were excluded. LOS was dichotomized around the national median into early and late discharge. Hospital variation was investigated using (case-mix corrected) funnel plots. The association of LOS with patient, tumor, and treatment characteristics and 30-day readmission were investigated using multilevel multivariable logistic regression analyses.

**Results:**

Between 2019 and 2023, a total of 1,761 gastrectomies were performed, of which 1,235 had an uncomplicated postoperative course. The national median LOS was 5 days (IQR:4–6), ranging from 3 to 7 days among the 14 Dutch gastrectomy centers. After case-mix correction, one hospital had significantly higher and three had lower early discharge rates. For total and subtotal gastrectomy separately, only one hospital had significantly lower early discharge rates. Open surgery (OR:8.43; 95%CI:4.75–15.0) and age > 75 years (OR:1.72; 95%CI:1.17–2.51) were associated with prolonged LOS (> 5 days), while subtotal gastrectomy (OR:0.24; 95%CI:0.16–0.36) was associated with early discharge (≤ 5 days). The 30-day readmission rate was 6.0%, with no association with early discharge. When including complicated patients, postoperative complications were the most dominant factor of late discharge, both minor (OR:7.7; 95%CI:4.9–12.1) and severe complications (OR:9.6; 95%CI:6.7–13.8).

**Conclusions:**

Significant hospital variation in LOS after uncomplicated gastrectomy was found in the Netherlands. The primary factors affecting LOS were surgical approach (open vs. minimally invasive) and type of gastrectomy performed (total vs. subtotal). Early discharge did not increase readmission risk.

**Graphical abstract:**

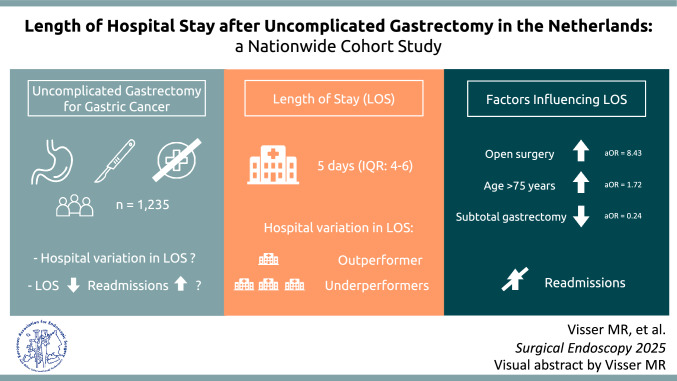

**Supplementary Information:**

The online version contains supplementary material available at 10.1007/s00464-025-12103-7.

Gastric cancer is currently the fifth most prevalent cancer globally [1]. Between 2018 and 2022, the lethality decreased from the third to the fifth most deadly type of cancer [1]. Incidence rates vary widely between countries, especially when comparing Eastern versus Western countries. Treatment strategies also vary worldwide, although gastrectomy is still the cornerstone of potentially curative treatment [2, 3]. Gastrectomy is a complex surgical procedure associated with significant postoperative morbidity and mortality, with reported rates of 11% to 46% and 1% to 7%, respectively [4–7]. Subsequent length of hospital stay (LOS) is substantial but also varies across the world and across healthcare systems [8–11]. The recently published international benchmark indicates LOS should be ≤ 11 days after total gastrectomy and ≤ 10 days after subtotal gastrectomy [12]

In 2011, the Dutch Upper Gastrointestinal Cancer Audit (DUCA) was established with the aim of monitoring and improving the quality of care of esophagogastric cancer surgery in the Netherlands [13]. By providing surgical teams with benchmarked, hospital-specific results, the goal is to reduce hospital variation in the Netherlands and enhance outcomes compared to international standards. One noticeable example is that since inception, the textbook outcome rate after gastrectomy, a measure for the ideal outcome, has risen from 32 to 55%, showcasing the improving quality of care [4, 14]. Similarly, a decreasing LOS was seen, probably leading to lower hospital expenses [4].

Extended LOS and complications are burdensome for both patients and hospital finances [15–19]. In-hospital stay after surgery is significantly impacted by postoperative complications [15, 19]. Minimizing these complications remains a key factor in shortening LOS. However, as 60%–70% of patients do not experience postoperative complications following gastrectomy, identifying factors associated with prolonged LOS after uncomplicated gastrectomy could help in reducing LOS [4, 20]. Studies investigating this association in gastrectomy patients are lacking. This study aimed to investigate hospital variation in LOS and its association with readmission rates after uncomplicated gastrectomy in the Netherlands.

## Materials and methods

This nationwide, population-based cohort study retrieved data from the Dutch Upper Gastrointestinal Cancer Audit (DUCA). This mandatory audit registers all patients having undergone surgery with the intent to resect esophageal and gastric cancer in the Netherlands since 2011. Postoperative outcomes (like morbidity, mortality and readmissions) are registered until 30 days after surgery or discharge. Data verification showed high completeness and accuracy of the data [21]. The current study was reviewed and approved by the DUCA scientific committee (DUCA2021-129).

### Patients

All patients who underwent intended curative gastrectomy for gastric or gastroesophageal junction cancer from 1-1-2019 until 31-12-2023 were considered for inclusion. Hospitals (4) that terminated performing gastrectomy for cancer during the study period were excluded from the study. Patients with per/postoperative complications according to the Gastric Complications Consensus Group were excluded [7]. Lastly, patients with missing LOS were excluded from analysis.

### Endpoints

The primary endpoint was hospital variation in LOS after uncomplicated gastrectomy. Secondary endpoints included patient, tumor, and treatment characteristics associated with LOS and the association between LOS and 30-day readmissions (any reason).

### Statistical analysis

The methods used in this study have previously been described by Voeten et al. [22]. LOS was calculated by subtracting the day of surgery from the day of discharge. Median LOS was reported on a national and hospital level, and for total and subtotal gastrectomy separately. Hospital variation was assessed using a boxplot. Hospitals were classified as outliers if their median LOS fell outside the bootstrapped 95% confidence intervals (10.000 samples) adjusted for their hospital case volume [23]. LOS was dichotomized around the national median into an early and late discharge group, with the exact median included in the early discharge group. The hospital complication rate over the study period was calculated per hospital and was similarly dichotomized around the median complication rate. A case-mix corrected funnel plot, correcting for the patient and tumor characteristics shown in Table S1, was used to assess hospital variation in early discharge [23, 24].

Patient, tumor, and treatment characteristics of patients with early and late discharge were compared using Chi-square, Wilcoxon rank sum, or Fisher’s exact tests. The characteristics and categories are shown in Table S1. Univariable and multilevel multivariable regression analyses were used to assess factors associated with late discharge. Factors with a p value < 0.1 in univariable analyses were included in the multilevel model to account for potential confounders that, while not statistically significant, could still influence the outcome. To adjust for potential unmeasured hospital differences, hospital ID was added as a random effect factor within the multilevel multivariable model. After adding the dichotomized LOS to the analyses, these analyses were repeated with 30-day readmission as the dependent variable to assess the impact of LOS on 30-day readmissions. Due to insufficient degrees of freedom, only factors with a p value < 0.05 were included in the multivariable analysis. All two-sided p values < 0.05 were considered statistically significant. Data analysis was performed using R-studio version 2024.04.1, The R Foundation for Statistical Computing [25].

### Additional analyses

To assess the hypothesis that LOS is significantly affected by complications, analyses were repeated including all gastrectomy patients, with and without complications. Median LOS was reported for the whole cohort, patients with postoperative complications and severe complications (Clavien–Dindo grade IIIa or higher). Similarly, factors associated with late discharge in the total cohort were assessed. Postoperative complications were added in these analyses as an independent factor.

## Results

**Fig. 1:**
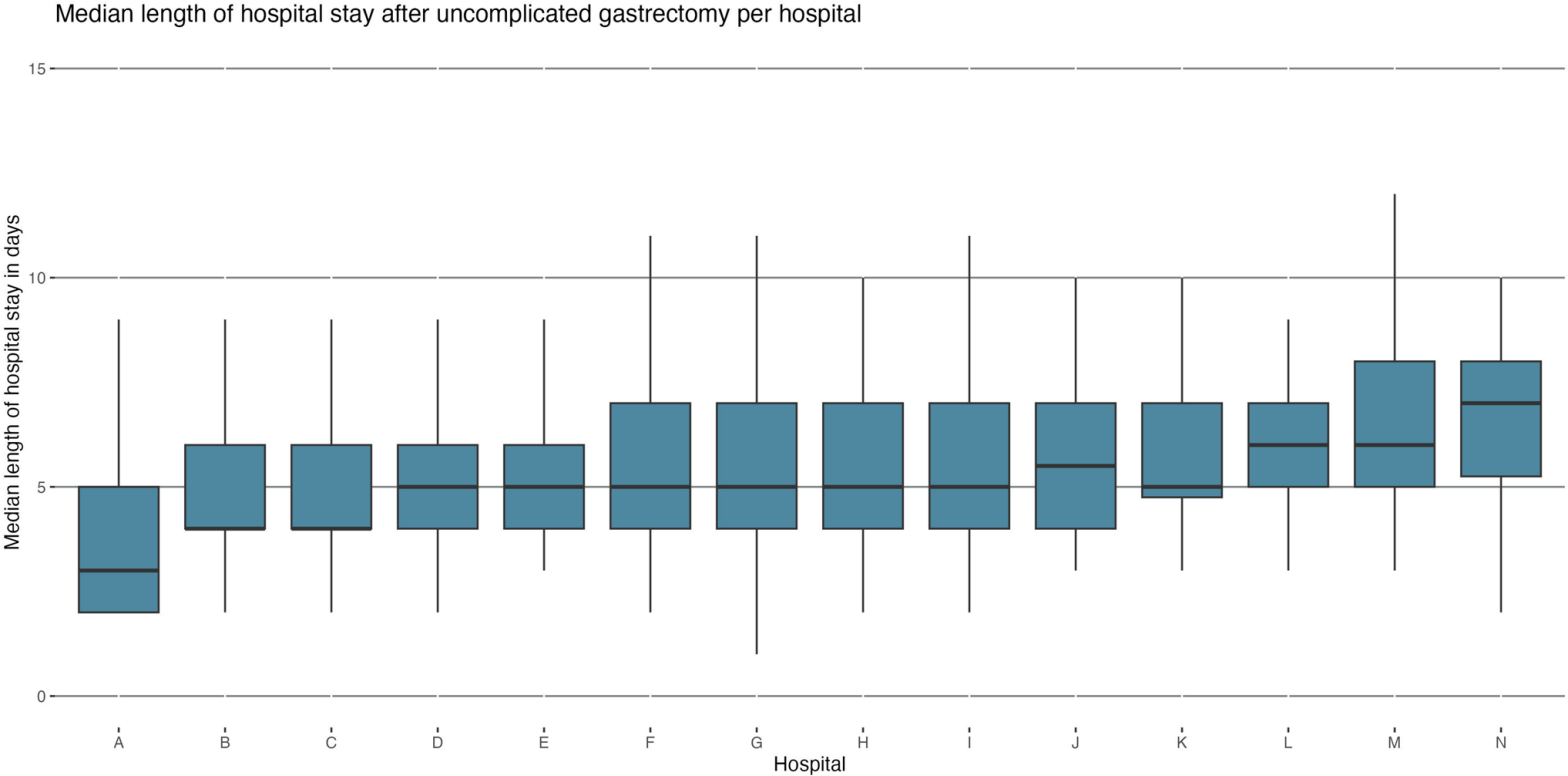
Median length of stay after uncomplicated gastrectomy per hospital between 2019 and 2023 in the Netherlands

Between 2019 and 2023, 1,761 patients with gastric or gastroesophageal junction cancer underwent gastrectomy in the Netherlands, of whom 1,237 went uncomplicated in 14 hospitals. Two patients had missing data on length of stay and were excluded. A flowchart of included patients is shown in Table S1. The national median LOS after uncomplicated gastrectomy was 5 days (IQR 4–7), ranging from 3 (IQR 2–5) to 7 days (IQR 5–8) among the 14 hospitals (Fig. 1). Three hospitals had a significantly lower median LOS, and two hospitals had a significantly higher median LOS. From 2019 to 2020, the national median LOS per year decreased from 6 days (IQR 4–7) to 5 days (IQR 3–7) and thereafter remained unchanged until 2023. For total gastrectomy (*n =* 518), the national median LOS from 2019 to 2023 was 6 days (IQR 5–7), ranging from 4 days (IQR 4–6) to 8 days (IQR 7–9) among hospitals. For subtotal gastrectomy (*n =* 717), the national median LOS was 5 days (IQR 4–6) in 2019, decreasing to 4 days (IQR 3–5) in 2023. Median LOS ranged from 3 days (IQR 2–4) to 6 days (IQR 4–7) among hospitals.

**Fig. 2:**
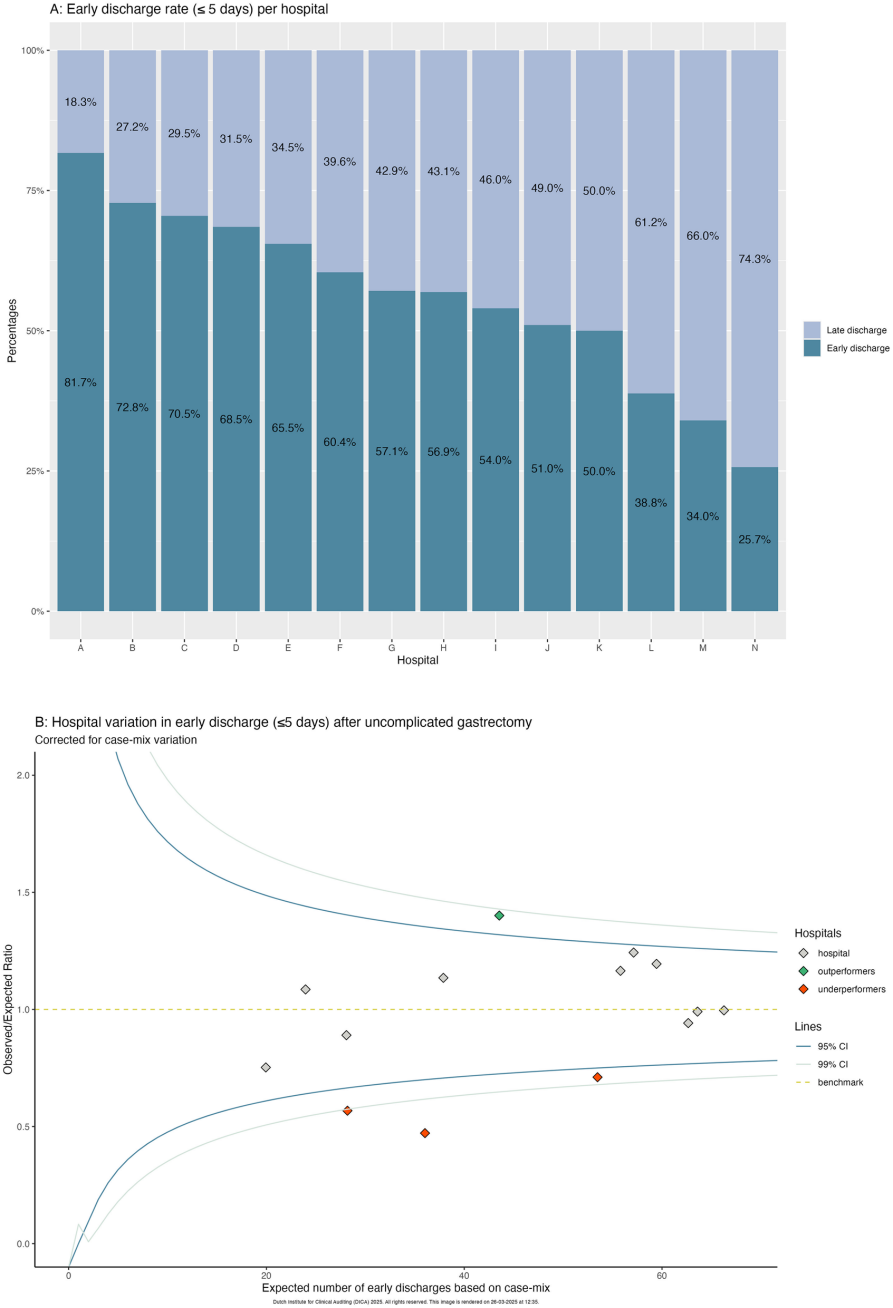

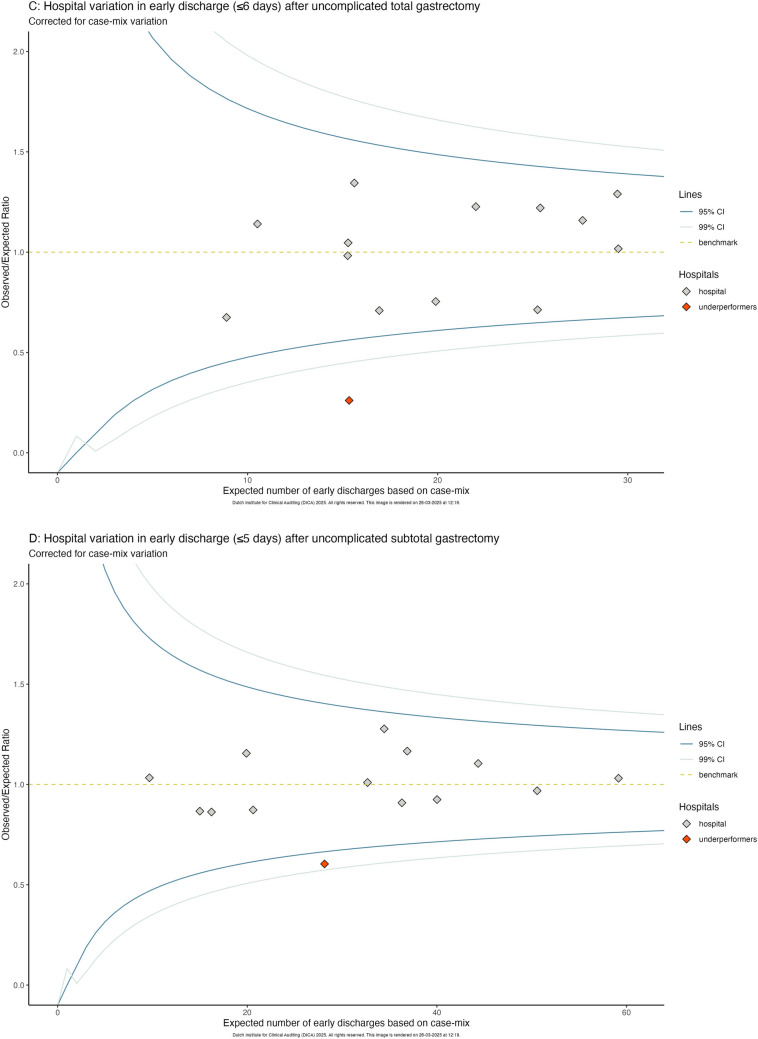
**A** Percentage of early discharge (≤ 5 days) per hospital. **B**-**D**: Funnel plot showing hospital variation in early discharge after (**B**) all gastrectomies (≤ 5 days), (**C**) total gastrectomies (≤ 6 days), (**D**) subtotal gastrectomies (≤ 5 days); corrected for case-mix (corrected for: sex, age, BMI, preoperative weight loss, history of esophageal/gastric surgery, Charlson Comorbidity Score, ASA score, tumor location, cT-stage, cN-stage)

After dichotomization around the national median (≤ 5 and > 5 days), 719 (58.2%) patients had an early discharge and 516 (41.8%) patients had a late discharge. The percentage of early discharge per hospital ranged from 25.7% to 81.7% (Fig. 2A). After correction for case-mix, one hospital had a significantly higher early discharge rate and three had a significantly lower early discharge rate (Fig. 2B). When analyzing total and subtotal gastrectomies separately, only one hospital had significantly lower early discharge rates for both procedures (Fig. 2C-D).

Patient, tumor, and treatment characteristics of patients with early and late discharge after uncomplicated gastrectomy are shown in Table 1. The groups differed significantly in previous esophageal and/or gastric surgery, tumor location, cT-stage, cM stage, type of gastrectomy, surgical approach, hospital volume, and hospital complication rate. In multilevel multivariable logistic regression analyses, age (> 75 years) and open surgery were associated with late discharge, while subtotal gastrectomy was associated with early discharge (Table 2). After correction for case-mix, including surgical approach and type of gastrectomy, one hospital had a significantly lower early discharge rate (Fig. S2). The national median LOS was 7 days (IQR 6–8) for open and 5 days (IQR 4–6) for minimally invasive surgery. Patient, tumor, and treatment characteristics of the open and minimally invasive surgery groups are shown in Table S4.

**Table 1:** Patient, tumor, and treatment characteristics of patients with early (≤ 5 days) and late discharge (> 5 days) after uncomplicated gastrectomy in 2019–2023

Characteristic	Overall, *N =* 1,235^a^	Late discharge (> 5 days), *N =* 516^a^	Early discharge (≤ 5 days), *N =* 719^a^	p value^b^
Sex				0.966
Male	747 (61)	312 (60)	435 (61)	
Female	487 (39)	204 (40)	283 (39)	
Age (median in years)	70 [62, 77]	70 [60, 77]	71 [63, 77]	0.262
Age categories				0.103
< 65 years	408 (33)	185 (36)	223 (31)	
65 to 75 years	428 (35)	163 (32)	265 (37)	
> 75 years	399 (32)	168 (33)	231 (32)	
BMI				0.923
< 20 kg	85 (6.9)	39 (7.6)	46 (6.4)	
20–25 kg	640 (52)	262 (51)	378 (53)	
26–30 kg	367 (30)	156 (30)	211 (29)	
> 30 kg	135 (11)	56 (11)	79 (11)	
Missing	8 (0.6)	3 (0.6)	5 (0.7)	
Preoperative weight loss				0.207
No weight loss	404 (33)	153 (30)	251 (35)	
1–5 kg	339 (27)	144 (28)	195 (27)	
6–10 kg	241 (20)	107 (21)	134 (19)	
> 10 kg	134 (11)	65 (13)	69 (9.6)	
Missing	117 (9.5)	47 (9.1)	70 (9.7)	
ASA score				0.338
1–2	692 (56)	277 (54)	415 (58)	
3 +	535 (43)	236 (46)	299 (42)	
Missing	8 (0.6)	3 (0.6)	5 (0.7)	
Charlson Comorbidity Score				0.788
0	478 (39)	205 (40)	273 (38)	
1	309 (25)	125 (24)	184 (26)	
2 +	448 (36)	186 (36)	262 (36)	
Esophageal or gastric surgery in medical history				**0.032**
No	1,178 (95)	485 (94)	693 (96)	
Yes	55 (4.5)	31 (6.0)	24 (3.3)	
Unknown/Missing	2 (0.2)	0 (0)	2 (0.3)	
Tumor location				** < 0.001**
GEJ	92 (7.4)	60 (12)	32 (4.5)	
Fundus	79 (6.4)	54 (10)	25 (3.5)	
Corpus	383 (31)	170 (33)	213 (30)	
Antrum/Pylorus	601 (49)	185 (36)	416 (58)	
Total stomach	47 (3.8)	26 (5.0)	21 (2.9)	
Rest stomach/anastomosis	20 (1.6)	14 (2.7)	6 (0.8)	
Unknown location	13 (1.1)	7 (1.4)	6 (0.8)	
Clinical tumor stage				**0.003**
T0-2	340 (28)	135 (26)	205 (29)	
T3	639 (52)	256 (50)	383 (53)	
T4	125 (10)	72 (14)	53 (7.4)	
Tx	130 (11)	53 (10)	77 (11)	
Missing	1 (< 0.1)	0 (0)	1 (0.1)	
Clinical node stage				0.114
N0	724 (59)	286 (55)	438 (61)	
N +	459 (37)	210 (41)	249 (35)	
Nx	51 (4.1)	20 (3.9)	31 (4.3)	
Missing	1 (< 0.1)	0 (0)	1 (0.1)	
Clinical metastasis stage				**0.039**
M0	1,167 (94)	479 (93)	688 (96)	
M +	16 (1.3)	11 (2.1)	5 (0.7)	
Mx	52 (4.2)	26 (5.0)	26 (3.6)	
Neoadjuvant therapy				0.493
Chemotherapy	742 (60)	308 (60)	434 (60)	
None	414 (34)	171 (33)	243 (34)	
Chemoradiotherapy	76 (6.2)	36 (7.0)	40 (5.6)	
Radiotherapy	1 (< 0.1)	1 (0.2)	0 (0)	
Missing	2 (0.2)	0 (0)	2 (0.3)	
Type of gastrectomy				** < 0.001**
Total gastrectomy	518 (42)	313 (61)	205 (29)	
Subtotal gastrectomy	717 (58)	203 (39)	514 (71)	
Surgical approach				** < 0.001**
Open	178 (14)	141 (27)	37 (5.1)	
Minimally invasive	1,057 (86)	375 (73)	682 (95)	
Annual hospital gastrectomy volume				**0.043**
< 30	669 (54)	297 (58)	372 (52)	
≥ 30	566 (46)	219 (42)	347 (48)	
Hospital complication rate				**0.045**
≤ national median	735 (60)	290 (56)	445 (62)	
> national median	500 (40)	226 (44)	274 (38)	

Bold data indicates a p-value < 0.05

^a^n (%); Median [IQR]

^b^Pearson's Chi-squared test; Wilcoxon rank sum test; Fisher's exact test

**Table 2:** Univariable and multilevel multivariable logistic regression analyses of factors associated with late discharge (> 5 days) after uncomplicated gastrectomy in 2019–2023

			Univariable analysis			Multivariable analysis	
Characteristic	N	OR	CI^**a**^ (95%)	p value	aOR^**a**^	CI^**a**^ (95%)	p value
Sex	1,234						
Male		1					
Female		1.01	0.80–1.27	0.966			
Age categories	1,235						
< 65 years		1			1		
65 to 75 years		0.74	0.56–0.98	**0.033**	1.04	0.73–1.50	0.814
> 75 years		0.88	0.66–1.16	0.354	1.72	1.17–2.51	**0.005**
Charlson Comorbidity Score	1,235						
0		1					
1		0.90	0.68–1.21	0.499			
2 +		0.95	0.73–1.23	0.673			
BMI	1,227						
20–25 kg		1					
< 20 kg		1.22	0.77–1.93	0.385			
26–30 kg		1.07	0.82–1.38	0.627			
> 30 kg		1.02	0.70–1.49	0.907			
Preoperative weight loss	1,118						
No weight loss		1			1		
1–5 kg		1.21	0.90–1.63	0.202	1.39	0.96–2.03	0.084
6–10 kg		1.31	0.95–1.81	0.102	1.48	0.98–2.24	0.061
> 10 kg		1.55	1.04–2.29	**0.030**	1.52	0.92–2.50	0.102
ASA score	1,227						
1–2		1					
3 +		1.18	0.94–1.49	0.151			
Esophageal or gastric surgery in medical history	1,233						
No		1			1		
Yes		1.85	1.07–3.21	**0.028**	0.81	0.35–1.89	0.626
Tumor location	1,222						
Corpus		1			1		
GEJ		2.35	1.47–3.81	** < 0.001**	1.50	0.81–2.79	0.198
Fundus		2.71	1.63–4.59	** < 0.001**	1.39	0.71–2.70	0.332
Antrum/Pylorus		0.56	0.43–0.73	** < 0.001**	0.99	0.67–1.45	0.952
Total stomach		1.55	0.85–2.88	0.158	0.65	0.29–1.47	0.300
Rest stomach/anastomosis		2.92	1.15–8.40	**0.031**	1.58	0.36–6.94	0.543
Clinical tumor stage	1,104						
T0-2		1			1		
T3		1.01	0.78–1.33	0.914	0.88	0.61–1.26	0.483
T4		2.06	1.36–3.14	** < 0.001**	1.35	0.77–2.34	0.293
Clinical node stage	1,183						
N0		1			1		
N +		1.29	1.02–1.64	**0.034**	1.03	0.74–1.42	0.880
Clinical metastasis stage	1,183						
M0		1			1		
M +		3.16	1.14–10.1	**0.034**	1.13	0.27–4.73	0.872
Neoadjuvant therapy	1,232						
Chemotherapy		1					
None		0.99	0.78–1.27	0.946			
Chemoradiotherapy		1.27	0.79–2.04	0.325			
Type of gastrectomy	1,235						
Total gastrectomy		1			1		
Subtotal gastrectomy		0.26	0.20–0.33	** < 0.001**	0.24	0.16–0.36	** < 0.001**
Surgical approach	1,235						
Minimally invasive		1			1		
Open		6.93	4.77–10.3	** < 0.001**	8.43	4.75–15.0	** < 0.001**
Annual hospital gastrectomy volume	1,235						
< 30		1			1		
≥ 30		0.79	0.63–0.99	**0.043**	0.90	0.64–1.27	0.554
Hospital complication rate	1,235						
≤ national median		1			1		
> national median		1.27	1.01–1.59	**0.045**	1.13	0.66–1.94	0.659

Bold data indicates a p-value < 0.05

^a^aOR = adjusted odds ratio, *CI* confidence interval

### Readmissions

Patient, tumor, and treatment characteristics of patients with and without a readmission are shown in Table 3. Of the 1,235 included patients, 11 had an unknown readmission status. In total, 74 of 1,224 (6.0%) patients were readmitted within 30 days after discharge. In multivariable logistic regression analyses, BMI > 30 kg and not having undergone neoadjuvant therapy increased the odds of a readmission (Table S2). LOS was not associated with increased readmission rates.

**Table 3:** Patient, tumor and treatment characteristics of patients readmitted within 30 days after discharge following uncomplicated gastrectomy in 2019–2023

Characteristic	Overall, *N =* 1,224^a^	No readmission, *N =* 1,150^a^	Readmission, *N =* 74^a^	p value^b^
Sex				0.826
Male	742 (61)	698 (61)	44 (59)	
Female	481 (39)	451 (39)	30 (41)	
Age (median in years)	70 [62, 77]	70 [61, 77]	71 [63, 78]	0.775
Age categories				0.984
< 65 years	408 (33)	384 (33)	24 (32)	
65 to 75 years	421 (34)	395 (34)	26 (35)	
> 75 years	395 (32)	371 (32)	24 (32)	
BMI				0.163
< 20 kg	84 (6.9)	79 (6.9)	5 (6.8)	
20–25 kg	633 (52)	600 (52)	33 (45)	
26–30 kg	364 (30)	343 (30)	21 (28)	
> 30 kg	135 (11)	120 (10)	15 (20)	
Missing	8 (0.7)	8 (0.7)	0 (0)	
Preoperative weight loss				0.160
No weight loss	400 (33)	372 (32)	28 (38)	
1–5 kg	336 (27)	320 (28)	16 (22)	
6–10 kg	241 (20)	230 (20)	11 (15)	
> 10 kg	132 (11)	125 (11)	7 (9.5)	
Missing	115 (9.4)	103 (9.0)	12 (16)	
ASA score				0.192
1–2	688 (56)	652 (57)	36 (49)	
3 +	528 (43)	491 (43)	37 (50)	
Missing	8 (0.7)	7 (0.6)	1 (1.4)	
Charlson Comorbidity Score				0.151
0	475 (39)	454 (39)	21 (28)	
1	306 (25)	283 (25)	23 (31)	
2 +	443 (36)	413 (36)	30 (41)	
Esophageal or gastric surgery in medical history				0.240
No	1,167 (95)	1,099 (96)	68 (92)	
Yes	55 (4.5)	49 (4.3)	6 (8.1)	
Unknown/Missing	2 (0.2)	2 (0.2)	0 (0)	
Tumor location				0.250
GEJ	91 (7.4)	86 (7.5)	5 (6.8)	
Fundus	78 (6.4)	75 (6.5)	3 (4.1)	
Corpus	377 (31)	349 (30)	28 (38)	
Antrum/Pylorus	598 (49)	563 (49)	35 (47)	
Total stomach	47 (3.8)	47 (4.1)	0 (0)	
Rest stomach/anastomosis	20 (1.6)	19 (1.7)	1 (1.4)	
Unknown location	13 (1.1)	11 (1.0)	2 (2.7)	
Clinical tumor stage				0.142
T0-2	336 (27)	312 (27)	24 (32)	
T3	634 (52)	604 (53)	30 (41)	
T4	124 (10)	117 (10)	7 (9.5)	
Tx	129 (11)	116 (10)	13 (18)	
Missing	1 (< 0.1)	1 (< 0.1)	0 (0)	
Clinical node stage				0.263
N0	716 (58)	673 (59)	43 (58)	
N +	456 (37)	431 (37)	25 (34)	
Nx	51 (4.2)	45 (3.9)	6 (8.1)	
Missing	1 (< 0.1)	1 (< 0.1)	0 (0)	
Clinical metastasis stage				**0.010**
M0	1,156 (94)	1,092 (95)	64 (86)	
M +	16 (1.3)	14 (1.2)	2 (2.7)	
Mx	52 (4.2)	44 (3.8)	8 (11)	
Neoadjuvant therapy				** < 0.001**
Chemotherapy	735 (60)	701 (61)	34 (46)	
None	411 (34)	375 (33)	36 (49)	
Chemoradiotherapy	75 (6.1)	73 (6.3)	2 (2.7)	
Radiotherapy	1 (< 0.1)	0 (0)	1 (1.4)	
Missing	2 (0.2)	1 (< 0.1)	1 (1.4)	
Type of gastrectomy				> 0.999
Total gastrectomy	512 (42)	481 (42)	31 (42)	
Subtotal gastrectomy	712 (58)	669 (58)	43 (58)	
Surgical approach				**0.032**
Open	177 (14)	160 (14)	17 (23)	
Minimally invasive	1,047 (86)	990 (86)	57 (77)	
Annual hospital gastrectomy volume				0.806
< 30	662 (54)	623 (54)	39 (53)	
≥ 30	562 (46)	527 (46)	35 (47)	
Length of hospital stay				0.081
Late discharge (> 5 days)	510 (42)	472 (41)	38 (51)	
Early discharge (≤ 5 days)	714 (58)	678 (59)	36 49)	

Bold data indicates a p-value < 0.05

^a^n (%); Median [IQR]

^b^Pearson's Chi-squared test; Wilcoxon rank sum test; Fisher's exact test

### Additional analysis: including complicated gastrectomies

Of the 2,745 patients with gastric or gastroesophageal junction cancer, 1,759 (64%) underwent gastrectomy (complicated and uncomplicated). The median LOS in this cohort was 6 days (IQR 4–9), decreasing from 7 to 6 days from 2019 to 2020 and thereafter remaining unchanged until 2023. Of these patients, 501 (28.5%) patients had postoperative complications, with 176 (10%) having minor complications (Clavien–Dindo grade I-II) and 325 (18.5%) having severe complications (≥ Clavien–Dindo grade IIIa). The median LOS was 11 days (IQR 7–21) in all patients with postoperative complications, 9 days (IQR 7–14) in patients with minor complications, and 15 days (IQR 8–28) in patients with severe complications. Factors associated with late discharge in all patients undergoing gastrectomy are shown in Table S3. These included age (> 75 years), 6-10 kg weight loss, total gastrectomy, open surgery, and postoperative complications. Postoperative complications were the most dominant factor of late discharge, both minor (OR:7.7; 95%CI:4.9–12.1) and severe complications (OR:9.6; 95%CI:6.7–13.8). The 30-day readmission rate in this cohort was 13.1%.

## Discussion

This nationwide cohort study, using data from all patients having undergone gastrectomy for gastric cancer in the Netherlands, showed significant hospital variation in LOS following uncomplicated gastrectomy. Hospital variation was observed for both total and subtotal gastrectomies, with one hospital having a consistently lower rate of early discharge for both types of procedures. Early discharge was not associated with an increased risk of readmissions after uncomplicated gastrectomy.

In the Netherlands, the national median length of hospital stay following uncomplicated gastrectomy was 5 days for all gastrectomies, 6 days for total and decreased to 4 days for subtotal gastrectomies in 2023. To our knowledge, no previous studies have addressed LOS following uncomplicated gastrectomy.

The primary factors affecting LOS in this study were surgical approach (open vs. laparoscopic surgery) and type of gastrectomy performed (total vs. subtotal). Total gastrectomy is a more complex and extensive procedure than subtotal gastrectomy and is linked to longer hospital stays, higher complication rates, and increased mortality [8, 12]. There is an ongoing debate regarding open versus laparoscopic surgery. The LOGICA trial, comparing laparoscopic and open gastrectomy, reported no differences in LOS between both procedures [8]. However, an analysis of the dissemination of laparoscopic gastrectomy in the Netherlands showed significant reductions in LOS following the LOGICA trial [26]. In this study, the open surgery group consisted of slightly more complex patients (Table S4), although these differences were adjusted for in the multivariable analysis. International series reported similar LOS benefits of laparoscopic gastrectomy [9]. As the use of robot-assisted gastrectomy continues to expand, the LOS could potentially decrease even further [10, 27]. These findings suggest that a minimally invasive approach is preferable to open gastrectomy for reducing LOS and, when possible, subtotal gastrectomy should be favored over total gastrectomy.

Hospital variation in LOS for total and subtotal gastrectomy separately (1 outlier) was more limited compared to the full cohort (4 outliers), which could be partially attributable to the lower case volume in these analyses. Centers that performed more total gastrectomies tended to have a higher overall LOS. One center had significantly lower early discharge rates in both total and subtotal gastrectomies, showing room for improvement for both procedures. As most centers performed within the benchmark for both total and subtotal gastrectomy, the need for improvement might however seem minimal [12]. In our view, the outcomes achieved by the top 25% best performing hospitals should serve as the target, rather than the overall mean of all hospitals. Three hospitals demonstrated that significantly better outcomes are possible, providing a target for care improvement of uncomplicated patients across centers. The absolute LOS difference between hospitals, 4 days for total and 3 days for subtotal gastrectomy, underscores this potential, which could benefit patient outcomes. As discussed above, opting for minimally invasive rather than open surgery, where institutional expertise exists, and, when oncological feasible, choosing subtotal over total gastrectomy are two concrete factors hospitals can target to reduce LOS. However, even after adjusting for these factors (Fig. S2), significant hospital variation remained. Older patients were more likely to experience prolonged LOS, possibly due to medical complexity but also organizational factors, such as delays in post-discharge care. Other factors, such as preoperative weight loss and comorbidities, were associated with prolonged LOS in univariable analysis, though not in the multivariable model. Nonetheless, these factors may still play a role in clinical decision-making when identifying patients suitable for early discharge. Since only uncomplicated patients were included, the variation in LOS is also likely influenced by differences in clinical care pathways and discharge logistics, areas that should be manageable across hospitals [22]. These care pathways impact process quality and, consequently, LOS, by influencing the timing of key steps such as extubation, intensive care unit discharge, and early mobilization [28–30]. Other factors, such as scheduling surgery to avoid weekend discharges, coordinating timely in-home care, and patient education of the perioperative course and discharge targets, also play a role. Through shared learning and straightforward adjustments to protocols or logistics, improving these factors should be feasible.

Another element of perioperative care believed to significantly influence LOS is early recovery after surgery (ERAS) protocols [31]. These protocols include aspects such as perioperative nutrition, analgesia, drains, and mobilization. In the full cohort, preoperative weight loss was associated with prolonged LOS (Table S3), an aspect targeted in ERAS protocols. Multiple studies reported significant reductions in LOS after the introduction of ERAS protocols [32–35]. Although results are difficult to compare due to heterogeneity between the ERAS protocols and regular care between studies, LOS seems to be one of the main beneficiaries of these protocols. The DUCA currently has no information on the use of ERAS protocols in different hospitals, although this might be incorporated in the audit in future registration years.

Readmission rates were 6% following uncomplicated gastrectomy. Since complications are an important predictor for readmissions, this rate was higher (13%) in the overall gastrectomy cohort [36]. The readmission rate is on the higher end compared to those reported in the literature (4–12%) and exceeds the benchmark [12]. Shortening LOS raises concerns about potentially higher readmission rates, as patients may require more time to recover in the controlled environment of the hospital. However, this study, along with others, found no such association [36, 37]. Similarly, a previous DUCA study investigating this association did not find increasing readmission rates when decreasing LOS after uncomplicated esophagectomy [22].

Two factors did increase the risk of readmission after uncomplicated gastrectomy in this study: a BMI > 30 and not being treated with neoadjuvant therapy. Surgery in obese patients is more complex than in non-obese patients. The Dutch guidelines recommend neoadjuvant chemotherapy for locally advanced gastric cancer [38]. Patients not receiving neoadjuvant therapy either undergo unplanned semi-acute surgery or are unfit to undergo intensive systemic therapy, possibly making them more prone to late complications causing readmissions.

Since inception of the DUCA in 2011, a decrease in in-hospital stay after all gastrectomies was seen from 10 to 7 days in 2018 [4]. This study showed a further decline to 6 days in 2020 after which it plateaued. Although the hospital variation indicates there is still room for improvement in certain centers, these findings may also suggest that the Netherlands as a country has approached the lower limits of in-hospital stay. Compared to an international cohort of 27 European expert centers (9 days) and the benchmarks established by Schneider et al. for total (≤ 11 days) and distal gastrectomy (≤ 10 days), both lengths of stay are already significantly shorter than the international standards [11, 12, 39]. However, a recent American study suggested that a further reduction of LOS to 1 or 2 days is safe and feasible. The authors reported a LOS decrease from 7 to 2 days in combination with a lower complication rate [40]. By introducing a pathway that included factors such as regionalization, minimally invasive surgery, and ERAS protocols, this relatively short LOS was reached, while at the same time increasing the use of neoadjuvant therapy and a higher D2-lymphadenectomy rate. However, the subtotal gastrectomy rate was higher compared to the current study (79% vs. 58%).

Since 2018, Dutch esophagogastric surgeons have been organizing yearly best-practice meetings [41, 42]. In 2023, outcomes of gastric cancer surgery were introduced in these meetings, including LOS after gastrectomy and uncomplicated gastrectomy, with the aim of reducing hospital variation and improving on a national level [11]. These meetings addressed factors such as patient education, clinical care pathways, discharge bottlenecks, and protocols. Comprehensive results in gastrectomy outcome due to these meetings are anticipated in the future.

With healthcare finances becoming a growing focus worldwide, there may be increasing attention to LOS. Recently, the Ministry of Health in the Netherlands released an agreement focused on improving the quality of care while managing rising healthcare costs [43]. Identifying methods to reduce complications while simultaneously shortening the LOS for uncomplicated procedures could help lower the costs of gastric cancer surgery [15–19].

This study has some limitations. The DUCA records complications and readmissions until 30 days after discharge, limiting the ability to conduct long-term analyses. Similarly, not all aspects of perioperative care are captured in the DUCA database, potentially leading to unmeasured confounding. Due to the relatively small number of readmitted patients, a multivariable analysis of influencing factors was limited. Furthermore, data on the use of ERAS protocols are lacking, limiting the interpretability of LOS variation following uncomplicated gastrectomy. Comparing LOS between countries is difficult because of differences in postoperative care and reimbursement structures across healthcare systems.

In conclusion, significant hospital variation in LOS can be found following uncomplicated gastrectomy in the Netherlands. The primary factors affecting LOS were surgical approach (open vs. laparoscopic surgery) and type of gastrectomy performed (total vs. subtotal). Including complicated gastrectomies, postoperative complications emerged as the predominant factor influencing LOS. Three hospitals had a significantly lower median LOS and could serve as the target for improvement. Reducing LOS did not increase the risk of 30-day readmissions. Optimization of clinical care pathways and discharge logistics should further shorten LOS following uncomplicated gastrectomy, potentially reducing both patient burden and hospital finances.

## Supplementary Information

Below is the link to the electronic supplementary material.

Supplementary file1 (DOCX 928 KB)
