# Supplementary material for: Length of hospital stay after uncomplicated gastrectomy in the Netherlands: a nationwide cohort study
Source: Surg Endosc. 2025 Oct 6;40(1):159–73. doi: 10.1007/s00464-025-12103-7 (PMC12823739; doi:10.1007/s00464-025-12103-7)
Supplement: Supplementary file 1 — Supplementary file1 (DOCX 928 KB) [file 464_2025_12103_MOESM1_ESM.docx]

# SUPPLEMENTARIES


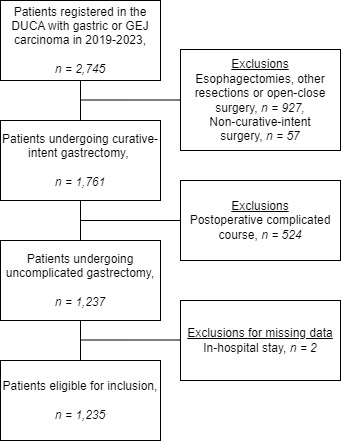


**Figure S1: Flowchart of included patients.**

| **Table S1: Variables included for analysis.** | |
| --- | --- |
| **Variable** | **Categories** |
| *Sex* | Male, Female |
| *Age* | < 65, 65 – 75, > 75 |
| *Charlson Comorbidity score* | 0, 1, 2 + |
| *BMI (kg/m^2^)* | < 20, 20 – 25, 26 – 30, > 30 |
| *Preoperative weight loss (kg)* | No weight loss, 1 – 5, 6 – 10, > 10 |
| *ASA score* | 1 – 2, 3 + |
| *Previous esophageal or gastric surgery* | Yes, No |
| *Tumor location* | Gastro-esophageal junction (GEJ), Fundus, Corpus, Antrum/Pylorus, Total stomach, Rest stomach/anastomosis |
| *Clinical tumor stage* | T0 – 2, T3, T4 |
| *Clinical node stage* | N0, N+ |
| *Clinical metastasis stage* | M0, M+ |
| *Neoadjuvant therapy* | Chemotherapy, None, Chemoradiotherapy |
| *Type of gastrectomy* | Total gastrectomy, subtotal gastrectomy |
| *Surgical approach* | Minimally invasive, open |
| *Hospital volume (the annual total gastrectomy hospital volume was assigned to each patient and thereafter dichotomized into <30 or ≥30)* | *<30, ≥30* |

Figure S2: Funnel plot showing hospital variation in early discharge (≤5 days) after uncomplicated gastrectomy; corrected for case-mix including treatment characteristics (corrected for: sex, age, BMI, preoperative weight loss, history of esophageal/gastric surgery, Charlson Comorbidity Score, ASA-score, tumor location, cT-stage, cN-stage, surgical approach and type of gastrectomy).


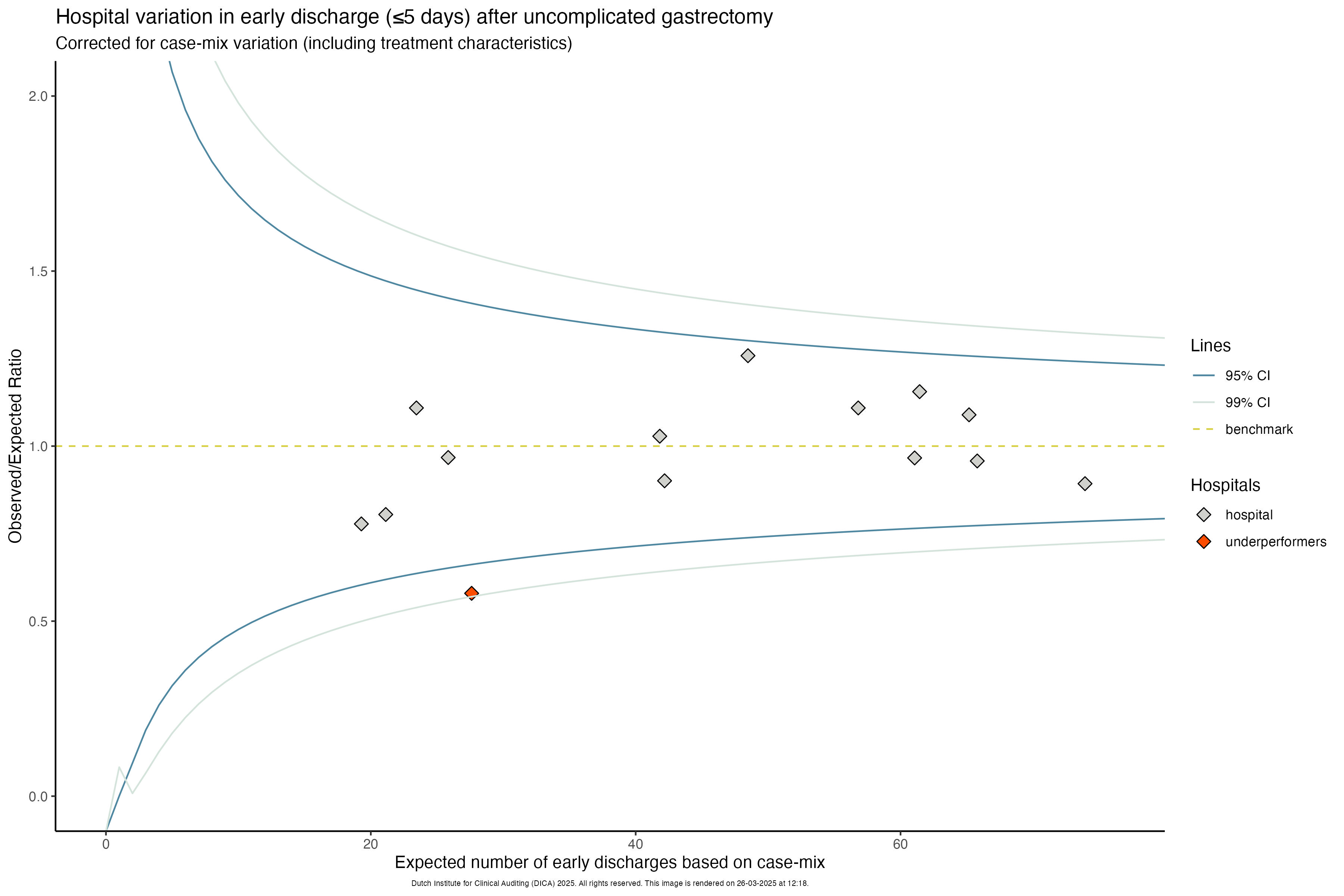


| **Table S2. Univariable and multilevel multivariable logistic regression analyses of factors associated with 30-day readmissions after uncomplicated gastrectomy in 2019-2023.** | | | | | | | |
| --- | --- | --- | --- | --- | --- | --- | --- |
|  |  |  | **Univariable analyses** | |  | **Multivariable analyses** | |
| **Characteristic** | **N** | **OR** | **CI^a^ (95%)** | **p-value** | **aOR^a^** | **CI^a^ (95%)** | **p-value** |
| **Sex** | 1,223 |  |  |  |  |  |  |
| Male |  | 1 |  |  |  |  |  |
| Female |  | 1.06 | 0.65-1.70 | 0.826 |  |  |  |
| **Age categories** | 1,224 |  |  |  |  |  |  |
| < 65 years |  | 1 |  |  |  |  |  |
| 65 to 75 years |  | 1.05 | 0.59-1.88 | 0.859 |  |  |  |
| > 75 years |  | 1.04 | 0.58-1.86 | 0.908 |  |  |  |
| **Charlson Comorbidity Score** | 1,224 |  |  |  |  |  |  |
| 0 |  | 1 |  |  |  |  |  |
| 1 |  | 1.76 | 0.95-3.25 | 0.070 |  |  |  |
| 2+ |  | 1.57 | 0.89-2.82 | 0.123 |  |  |  |
| **BMI** | 1,216 |  |  |  |  |  |  |
| 20-25 kg |  | 1 |  |  | 1 |  |  |
| < 20 kg |  | 1.15 | 0.39-2.79 | 0.776 | 1.21 | 0.40-2.96 | 0.702 |
| 26-30 kg |  | 1.11 | 0.63-1.94 | 0.709 | 1.09 | 0.60-1.93 | 0.765 |
| > 30 kg |  | 2.27 | 1.17-4.24 | **0.012** | 2.31 | 1.18-4.36 | **0.012** |
| **Preoperative weight loss** | 1,109 |  |  |  |  |  |  |
| No weight loss |  | 1 |  |  |  |  |  |
| 1-5 kg |  | 0.66 | 0.35-1.24 | 0.205 |  |  |  |
| 6-10 kg |  | 0.64 | 0.30-1.27 | 0.215 |  |  |  |
| > 10 kg |  | 0.74 | 0.29-1.66 | 0.497 |  |  |  |
| **ASA score** | 1,216 |  |  |  |  |  |  |
| 1-2 |  | 1 |  |  |  |  |  |
| 3+ |  | 1.36 | 0.85-2.20 | 0.198 |  |  |  |
| **Esophageal or Gastric surgery in medical history** | 1,222 |  |  |  |  |  |  |
| No |  | 1 |  |  |  |  |  |
| Yes |  | 1.98 | 0.74-4.45 | 0.129 |  |  |  |
| **Tumor location** | 1,211 |  |  |  |  |  |  |
| Corpus |  | 1 |  |  |  |  |  |
| GEJ |  | 0.72 | 0.24-1.78 | 0.520 |  |  |  |
| Fundus |  | 0.50 | 0.12-1.45 | 0.262 |  |  |  |
| Antrum/Pylorus |  | 0.77 | 0.46-1.30 | 0.331 |  |  |  |
| Total stomach |  | 0.00 | 0.00-935 | 0.979 |  |  |  |
| Rest stomach/anastomosis |  | 0.66 | 0.04-3.35 | 0.687 |  |  |  |
| **Clinical Tumor Stage** | 1,094 |  |  |  |  |  |  |
| T0-2 |  | 1 |  |  |  |  |  |
| T3 |  | 0.65 | 0.37-1.13 | 0.122 |  |  |  |
| T4 |  | 0.78 | 0.30-1.77 | 0.571 |  |  |  |
| **Clinical Node Stage** | 1,172 |  |  |  |  |  |  |
| N0 |  | 1 |  |  |  |  |  |
| N+ |  | 0.91 | 0.54-1.50 | 0.709 |  |  |  |
| **Clinical Metastasis Stage** | 1,172 |  |  |  |  |  |  |
| M0 |  | 1 |  |  |  |  |  |
| M+ |  | 2.44 | 0.38-8.97 | 0.245 |  |  |  |
| **Neoadjuvant therapy** | 1,221 |  |  |  |  |  |  |
| Chemotherapy |  | 1 |  |  | 1 |  |  |
| None |  | 1.98 | 1.22-3.22 | **0.006** | 1.94 | 1.19-3.18 | **0.008** |
| Chemoradiotherapy |  | 0.56 | 0.09-1.91 | 0.439 | 0.60 | 0.10-2.05 | 0.494 |
| **Type of gastrectomy** | 1,224 |  |  |  |  |  |  |
| Total gastrectomy |  | 1 |  |  |  |  |  |
| Subtotal gastrectomy |  | 1.00 | 0.62-1.62 | 0.991 |  |  |  |
| **Surgical approach** | 1,224 |  |  |  |  |  |  |
| Minimally invasive |  | 1 |  |  | 1 |  |  |
| Open |  | 1.85 | 1.02-3.19 | **0.034** | 1.90 | 1.04-3.32 | **0.029** |
| **Annual hospital volume** | 1,224 |  |  |  |  |  |  |
| <30 |  | 1 |  |  |  |  |  |
| ≥30 |  | 1.06 | 0.66-1.70 | 0.806 |  |  |  |
| **Length of hospital stay** | 1,224 |  |  |  |  |  |  |
| Late discharge (>5 days) |  | 1 |  |  |  |  |  |
| Early discharge (≤5 days) |  | 0.66 | 0.41-1.06 | 0.083 |  |  |  |
| 1. aOR = Adjusted Odds Ratio, CI = Confidence Interval | | | | | | | |

| **Table S3. Univariable and multilevel multivariable logistic regression analyses of factors associated with late discharge (>6 days) after complicated and uncomplicated gastrectomy in 2019-2023.** | | | | | | | |
| --- | --- | --- | --- | --- | --- | --- | --- |
|  |  |  | Univariable analyses | |  | Multivariable analyses | |
| **Characteristic** | **N** | **OR** | **CI^a^ (95%)** | **p-value** | **aOR^a^** | **CI^a^ (95%)** | **p-value** |
| **Sex** | 1,759 |  |  |  |  |  |  |
| Male |  | 1 |  |  |  |  |  |
| Female |  | 0.95 | 0.79-1.16 | 0.648 |  |  |  |
| **Age categories** | 1,759 |  |  |  |  |  |  |
| < 65 years |  | 1 |  |  | 1 |  |  |
| 65 to 75 years |  | 1.11 | 0.88-1.41 | 0.388 | 1.34 | 0.97-1.90 | 0.073 |
| > 75 years |  | 1.27 | 1.01-1.61 | **0.043** | 1.67 | 1.17-2.37 | **0.005** |
| **Charlson Comorbidity Score** | 1,759 |  |  |  |  |  |  |
| 0 |  | 1 |  |  |  |  |  |
| 1 |  | 0.96 | 0.75-1.23 | 0.763 |  |  |  |
| 2+ |  | 1.16 | 0.93-1.43 | 0.190 |  |  |  |
| **BMI** | 1,744 |  |  |  |  |  |  |
| 20-25 kg |  | 1 |  |  |  |  |  |
| < 20 kg |  | 1.28 | 0.88-1.86 | 0.188 |  |  |  |
| 26-30 kg |  | 0.90 | 0.72-1.12 | 0.352 |  |  |  |
| > 30 kg |  | 0.98 | 0.71-1.34 | 0.886 |  |  |  |
| **Preoperative weight loss** | 1,597 |  |  |  |  |  |  |
| No weight loss |  | 1 |  |  | 1 |  |  |
| 1-5 kg |  | 1.37 | 1.07-1.76 | **0.012** | 1.37 | 0.99-1.94 | 0.060 |
| 6-10 kg |  | 1.57 | 1.19-2.05 | **0.001** | 1.77 | 1.22-2.55 | **0.002** |
| > 10 kg |  | 1.47 | 1.06-2.04 | **0.021** | 1.26 | 0.81-1.97 | 0.303 |
| **ASA score** | 1,743 |  |  |  |  |  |  |
| 1-2 |  | 1 |  |  | 1 |  |  |
| 3+ |  | 1.20 | 1.00-1.46 | **0.056** | 1.21 | 0.92-1.60 | 0.177 |
| **Esophageal or Gastric surgery in medical history** | 1,755 |  |  |  |  |  |  |
| No |  | 1 |  |  | 1 |  |  |
| Yes |  | 2.02 | 1.32-3.12 | **0.001** | 1.53 | 0.73-3.22 | 0.264 |
| **Tumor location** | 1,743 |  |  |  |  |  |  |
| Corpus |  | 1 |  |  | 1 |  |  |
| GEJ |  | 2.14 | 1.49-3.11 | **<0.001** | 1.33 | 0.79-2.25 | 0.280 |
| Fundus |  | 2.25 | 1.53-3.34 | **<0.001** | 1.34 | 0.79-2.28 | 0.273 |
| Antrum/Pylorus |  | 0.63 | 0.50-0.79 | **<0.001** | 0.94 | 0.66-1.33 | 0.729 |
| Total stomach |  | 1.32 | 0.80-2.17 | 0.274 | 0.55 | 0.27-1.11 | 0.095 |
| Rest stomach/anastomosis |  | 2.66 | 1.37-5.43 | **0.005** | 0.83 | 0.24-2.80 | 0.758 |
| **Clinical Tumor Stage** | 1,580 |  |  |  |  |  |  |
| T0-2 |  | 1 |  |  | 1 |  |  |
| T3-4 |  | 1.40 | 1.12-1.75 | **0.003** | 1.17 | 0.85-1.63 | 0.334 |
| **Clinical Node Stage** | 1,695 |  |  |  |  |  |  |
| N0 |  | 1 |  |  | 1 |  |  |
| N+ |  | 1.31 | 1.08-1.60 | **0.007** | 1.14 | 0.85-1.52 | 0.391 |
| **Clinical Metastasis Stage** | 1,694 |  |  |  |  |  |  |
| M0 |  | 1 |  |  | 1 |  |  |
| M+ |  | 4.62 | 2.18-10.9 | **<0.001** | 1.24 | 0.44-3.51 | 0.689 |
| **Neoadjuvant therapy** | 1,754 |  |  |  |  |  |  |
| Chemotherapy |  | 1 |  |  |  |  |  |
| None |  | 1.06 | 0.87-1.30 | 0.549 |  |  |  |
| Chemoradiotherapy |  | 1.06 | 0.71-1.57 | 0.778 |  |  |  |
| **Type of gastrectomy** | 1,759 |  |  |  |  |  |  |
| Total gastrectomy |  | 1 |  |  | 1 |  |  |
| Subtotal gastrectomy |  | 0.29 | 0.24-0.36 | **<0.001** | 0.38 | 0.27-0.53 | **<0.001** |
| **Surgical approach** | 1,759 |  |  |  |  |  |  |
| Minimally invasive |  | 1 |  |  | 1 |  |  |
| Open |  | 4.10 | 3.14-5.59 | **<0.001** | 4.16 | 2.64-6.54 | **<0.001** |
| **Annual hospital volume** | 1,759 |  |  |  |  |  |  |
| <30 |  | 1 |  |  |  |  |  |
| ≥30 |  | 0.94 | 0.78-1.14 | 0.531 |  |  |  |
| **Complications** | 1,759 |  |  |  |  |  |  |
| No |  | 1 |  |  | 1 |  |  |
| CD^b^ grade I-II |  | 8.03 | 5.61-11.7 | **<0.001** | 7.73 | 4.94-12.1 | **<0.001** |
| CD^b^ grade ≥IIIa |  | 10.7 | 7.94-14.6 | **<0.001** | 9.57 | 6.65-13.8 | **<0.001** |
| 1. aOR = Adjusted Odds Ratio, CI = Confidence Interval 2. CD = Clavien Dindo | | | | | | | |

| **Table 4. Patient, tumor and treatment characteristics of patients undergoing uncomplicated open and minimally invasive gastrectomy in 2019-2023.** | | | | | |
| --- | --- | --- | --- | --- | --- |
| **Characteristic** | **N** | **Overall**  N = 1,235^1^ | **Open**  N = 178^1^ | **Minimally Invasive**  N = 1,057^1^ | **p-value**^2^ |
| **Sex** | 1,234 |  |  |  | **0.035** |
| Male |  | 747 (61%) | 121 (68%) | 626 (59%) |  |
| Female |  | 487 (39%) | 57 (32%) | 430 (41%) |  |
| **Age (median in years)** | 1,235 | 70 [62, 77] | 67 [60, 75] | 71 [62, 78] | **<0.001** |
| **Age categories** | 1,235 |  |  |  | **0.002** |
| < 65 years |  | 408 (33%) | 78 (44%) | 330 (31%) |  |
| 65 to 75 years |  | 428 (35%) | 58 (33%) | 370 (35%) |  |
| > 75 years |  | 399 (32%) | 42 (24%) | 357 (34%) |  |
| **BMI** | 1,235 |  |  |  | 0.093 |
| < 20 kg |  | 85 (6.9%) | 9 (5.1%) | 76 (7.2%) |  |
| 20-25 kg |  | 640 (52%) | 86 (48%) | 554 (52%) |  |
| 26-30 kg |  | 367 (30%) | 67 (38%) | 300 (28%) |  |
| > 30 kg |  | 135 (11%) | 16 (9.0%) | 119 (11%) |  |
| Missing |  | 8 (0.6%) | 0 (0%) | 8 (0.8%) |  |
| **Preoperative weight loss** | 1,235 |  |  |  | 0.848 |
| No weight loss |  | 404 (33%) | 62 (35%) | 342 (32%) |  |
| 1-5 kg |  | 339 (27%) | 48 (27%) | 291 (28%) |  |
| 6-10 kg |  | 241 (20%) | 30 (17%) | 211 (20%) |  |
| > 10 kg |  | 134 (11%) | 19 (11%) | 115 (11%) |  |
| Missing |  | 117 (9.5%) | 19 (11%) | 98 (9.3%) |  |
| **ASA score** | 1,235 |  |  |  | 0.403 |
| 1-2 |  | 692 (56%) | 108 (61%) | 584 (55%) |  |
| 3+ |  | 535 (43%) | 69 (39%) | 466 (44%) |  |
| Missing |  | 8 (0.6%) | 1 (0.6%) | 7 (0.7%) |  |
| **Charlson Comorbidity Score** | 1,235 |  |  |  | 0.473 |
| 0 |  | 478 (39%) | 72 (40%) | 406 (38%) |  |
| 1 |  | 309 (25%) | 38 (21%) | 271 (26%) |  |
| 2 |  | 448 (36%) | 68 (38%) | 380 (36%) |  |
| **Esophageal or Gastric surgery in medical history** | 1,235 |  |  |  | **<0.001** |
| No |  | 1,178 (95%) | 160 (90%) | 1,018 (96%) |  |
| Yes |  | 55 (4.5%) | 18 (10%) | 37 (3.5%) |  |
| Unknown/Missing |  | 2 (0.2%) | 0 (0%) | 2 (0.2%) |  |
| **Tumor location** | 1,235 |  |  |  | **<0.001** |
| GEJ |  | 92 (7.4%) | 15 (8.4%) | 77 (7.3%) |  |
| Fundus |  | 79 (6.4%) | 21 (12%) | 58 (5.5%) |  |
| Corpus |  | 383 (31%) | 55 (31%) | 328 (31%) |  |
| Antrum/Pylorus |  | 601 (49%) | 64 (36%) | 537 (51%) |  |
| Total stomach |  | 47 (3.8%) | 14 (7.9%) | 33 (3.1%) |  |
| Rest stomach/anastomosis |  | 20 (1.6%) | 8 (4.5%) | 12 (1.1%) |  |
| Unknown location |  | 13 (1.1%) | 1 (0.6%) | 12 (1.1%) |  |
| **Clinical Tumor Stage** | 1,235 |  |  |  | **<0.001** |
| T0-2 |  | 340 (28%) | 41 (23%) | 299 (28%) |  |
| T3 |  | 639 (52%) | 77 (43%) | 562 (53%) |  |
| T4 |  | 125 (10%) | 33 (19%) | 92 (8.7%) |  |
| Tx |  | 130 (11%) | 27 (15%) | 103 (9.7%) |  |
| Missing |  | 1 (<0.1%) | 0 (0%) | 1 (<0.1%) |  |
| **Clinical Node Stage** | 1,235 |  |  |  | 0.492 |
| N0 |  | 724 (59%) | 101 (57%) | 623 (59%) |  |
| N+ |  | 459 (37%) | 66 (37%) | 393 (37%) |  |
| Nx |  | 51 (4.1%) | 11 (6.2%) | 40 (3.8%) |  |
| Missing |  | 1 (<0.1%) | 0 (0%) | 1 (<0.1%) |  |
| **Clinical Metastasis Stage** | 1,235 |  |  |  | **<0.001** |
| M0 |  | 1,167 (94%) | 156 (88%) | 1,011 (96%) |  |
| M+ |  | 16 (1.3%) | 9 (5.1%) | 7 (0.7%) |  |
| Mx |  | 52 (4.2%) | 13 (7.3%) | 39 (3.7%) |  |
| **Neoadjuvant therapy** | 1,235 |  |  |  | 0.770 |
| Chemotherapy |  | 742 (60%) | 106 (60%) | 636 (60%) |  |
| None |  | 414 (34%) | 64 (36%) | 350 (33%) |  |
| Chemoradiotherapy |  | 76 (6.2%) | 8 (4.5%) | 68 (6.4%) |  |
| Radiotherapy |  | 1 (<0.1%) | 0 (0%) | 1 (<0.1%) |  |
| Missing |  | 2 (0.2%) | 0 (0%) | 2 (0.2%) |  |
| **Type of gastrectomy** | 1,235 |  |  |  | 0.052 |
| Total gastrectomy |  | 518 (42%) | 87 (49%) | 431 (41%) |  |
| Subtotal gastrectomy |  | 717 (58%) | 91 (51%) | 626 (59%) |  |
| **Annual hospital volume** | 1,235 |  |  |  | **0.050** |
| <30 |  | 669 (54%) | 109 (61%) | 560 (53%) |  |
| ≥30 |  | 566 (46%) | 69 (39%) | 497 (47%) |  |
| **Hospital complication rate** | 1,235 |  |  |  | 0.796 |
| < national median |  | 735 (60%) | 108 (61%) | 627 (59%) |  |
| > national median |  | 500 (40%) | 70 (39%) | 430 (41%) |  |
| **Length of stay** | 1,235 |  |  |  | **<0.001** |
| Long admission (>5 days) |  | 516 (42%) | 141 (79%) | 375 (35%) |  |
| Short admission (≤5 days) |  | 719 (58%) | 37 (21%) | 682 (65%) |  |
| ^1^n (%); Median [IQR] | | | | | |
| ^2^Pearson's Chi-squared test; Wilcoxon rank sum test | | | | | |

STROBE Statement—Checklist of items that should be included in reports of ***cohort studies***

|  | Item No | Recommendation |
| --- | --- | --- |
| **Title and abstract** | 1 | (*a*) Indicate the study’s design with a commonly used term in the title or the abstract |
|  |  | (*b*) Provide in the abstract an informative and balanced summary of what was done and what was found |
| Introduction | | |
| Background/rationale | 2 | Explain the scientific background and rationale for the investigation being reported |
| Objectives | 3 | State specific objectives, including any prespecified hypotheses |
| Methods | | |
| Study design | 4 | Present key elements of study design early in the paper |
| Setting | 5 | Describe the setting, locations, and relevant dates, including periods of recruitment, exposure, follow-up, and data collection |
| Participants | 6 | (*a*) Give the eligibility criteria, and the sources and methods of selection of participants. Describe methods of follow-up |
|  |  | (*b*) For matched studies, give matching criteria and number of exposed and unexposed |
| Variables | 7 | Clearly define all outcomes, exposures, predictors, potential confounders, and effect modifiers. Give diagnostic criteria, if applicable |
| Data sources/ measurement | 8* | For each variable of interest, give sources of data and details of methods of assessment (measurement). Describe comparability of assessment methods if there is more than one group |
| Bias | 9 | Describe any efforts to address potential sources of bias |
| Study size | 10 | Explain how the study size was arrived at |
| Quantitative variables | 11 | Explain how quantitative variables were handled in the analyses. If applicable, describe which groupings were chosen and why |
| Statistical methods | 12 | (*a*) Describe all statistical methods, including those used to control for confounding |
|  |  | (*b*) Describe any methods used to examine subgroups and interactions |
|  |  | (*c*) Explain how missing data were addressed |
|  |  | (*d*) If applicable, explain how loss to follow-up was addressed |
|  |  | (*e*) Describe any sensitivity analyses |
| Results | | |
| Participants | 13* | (a) Report numbers of individuals at each stage of study—eg numbers potentially eligible, examined for eligibility, confirmed eligible, included in the study, completing follow-up, and analysed |
|  |  | (b) Give reasons for non-participation at each stage |
|  |  | (c) Consider use of a flow diagram |
| Descriptive data | 14* | (a) Give characteristics of study participants (eg demographic, clinical, social) and information on exposures and potential confounders |
|  |  | (b) Indicate number of participants with missing data for each variable of interest |
|  |  | (c) Summarise follow-up time (eg, average and total amount) |
| Outcome data | 15* | Report numbers of outcome events or summary measures over time |
| Main results | 16 | (*a*) Give unadjusted estimates and, if applicable, confounder-adjusted estimates and their precision (eg, 95% confidence interval). Make clear which confounders were adjusted for and why they were included |
|  |  | (*b*) Report category boundaries when continuous variables were categorized |
|  |  | (*c*) If relevant, consider translating estimates of relative risk into absolute risk for a meaningful time period |
| Other analyses | 17 | Report other analyses done—eg analyses of subgroups and interactions, and sensitivity analyses |
| Discussion | | |
| Key results | 18 | Summarise key results with reference to study objectives |
| Limitations | 19 | Discuss limitations of the study, taking into account sources of potential bias or imprecision. Discuss both direction and magnitude of any potential bias |
| Interpretation | 20 | Give a cautious overall interpretation of results considering objectives, limitations, multiplicity of analyses, results from similar studies, and other relevant evidence |
| Generalisability | 21 | Discuss the generalisability (external validity) of the study results |
| Other information | | |
| Funding | 22 | Give the source of funding and the role of the funders for the present study and, if applicable, for the original study on which the present article is based |

*Give information separately for exposed and unexposed groups.
